# Les maladies chroniques non transmissibles chez les militaires sénégalais: étude transversale en 2013

**DOI:** 10.11604/pamj.2015.22.59.4777

**Published:** 2015-09-22

**Authors:** Ndiaye Abdoul Aziz, Seck Sidy Mohamed, Tall Alioune Badara, Gueye Boubacar, Sow Papa Gallo, Gaye Awa, Tal-Dia Anta

**Affiliations:** 1 Département de Santé Communautaire, Université de Bambey, Bambey, Sénégal; 2 Service de Santé des Armées Sénégalaises, Sénégal; 3 Département de Médecine Interne et Néphrologie, UFR des Sciences de la Santé, Université Gaston Berger de Saint-Louis, Sénégal; 4 Département Santé Publique, Université Cheikh Anta Diop, Dakar, Sénégal

**Keywords:** Epidémiologie, maladies non-transmissibles, militaires, Sénégal, Epidemiology, Non-communicable diseases, military, Senegal

## Abstract

**Introduction:**

Les maladies chroniques non transmissibles (MCNT) constituent un problème de santé publique. La transition épidémiologique coexiste avec les maladies infectieuses. En Afrique subsaharienne, leur ampleur est peu connue et l'OMS recommande aux pays à faible et moyen revenu de réaliser des enquêtes STEPS portant sur les comportements, des mesures physiques et biochimiques. L'absence de données au niveau national justifie cette étude auprès d'un groupe spécifique. L'objectif de l’étude était de déterminer la prévalence des MCNT et de leurs facteurs de risque chez les militaires Sénégalais.

**Méthodes:**

Une enquête transversale a été réalisée incluant les militaires âgés de 25 à 60 ans. La participation était volontaire et l'accord des autorités hiérarchiques a préalablement été obtenu. Un sondage stratifié à deux niveaux a été utilisé permettant d'avoir un échantillon ajusté de 1513 individus. Les données ont été saisies avec le logiciel EPI Info 6 et analysées à l'aide de R. Un score de risque a été déterminé sur la base de cinq facteurs.

**Résultats:**

Les résultats préliminaires concernent 1125 personnes. L’âge moyen était de 39,7 ±9,1 ans et le sex-ratio de 28,6. La prévalence du tabagisme actif était de 17,3% et ne variait pas significativement entre les différentes catégories d’âge. L’âge moyen auquel ils ont commencé à fumer était de 20,8 ±4,05 ans. La consommation médiane de fruits et légumes était de l'ordre de 4 par jour et seulement 5,7% des enquêtés prenaient au moins 5 portions par jour. Environs 72% des enquêtés avaient une activité physique intense ou modérée. Les prévalences de la surcharge pondérale, de l'HTA et du diabète étaient de 30,5%, 28,4% et 3,0% respectivement alors que la maladie rénale chronique était retrouvée chez un seul cas. Le calcul du score de risque cardiovasculaire a montré que 39,1% des militaires étaient à risque élevé (≥3 facteurs de risque) et que ce dernier augmentait avec l’âge.

**Conclusion:**

La prévalence élevée des MCNT dans ce groupe particulier laisse présager de l'ampleur dans la population générale. De ce point de vue, il est urgent de mettre en place un programme de prévention primaire et de dépistage pour anticiper les lourdes conséquences liées à ces maladies.

## Introduction

Les maladies chroniques non transmissibles (MCNT) constituent un problème prioritaire de santé publique. A l’échelle mondiale, la charge des maladies non transmissibles a augmenté rapidement. En 2001, elles représentaient pratiquement 60% des 56 millions de décès annuels et 47% de la charge de morbidité mondiale [1, 2]. Loin d’être l'exclusivité des pays riches, les MCNT constituent un lourd fardeau pour les pays pauvres [1, 3]. Le modèle de transition épidémiologique dans ces pays correspond plutôt à la coexistence des maladies infectieuses et des MCNT. En 2004, les estimations des causes de décès par région attribuent aux MCNT 25% en Afrique au Sud du Sahara, 48% en Asie du Sud, 63% au Moyen Orient et en Afrique du Nord et enfin, 87% pour les pays riches [3]. En Afrique, la morbidité des MCNT est en hausse [1, 3–5]. L’évolution de la fréquence du diabète illustre la progression des MCNT. En effet, en 1997, 63% des diabétiques vivaient dans les pays en développement, alors que cette affection était rare vingt ans auparavant; et l'OMS prévoit une proportion de 76% en 2025 [3, 6]. L'obésité, qui est un marqueur de la transition nutritionnelle, est en cours d'accroissement partout dans le monde. L'OMS parle d’épidémie mondiale qui affecte plus de 300 millions de personnes, dont 115 millions dans les pays en développement [7–9]. Le passage progressif d'un problème dominant de maigreur à un problème de surpoids et d'obésité est observé dans certains pays [7, 10].

La moitié des décès en ASS est attribuée aux maladies infectieuses, le quart aux MCNT. Certains auteurs prévoient que 46% de décès en ASS seront liés aux MCNT en 2030 [2, 11, 12]. L'accroissement des MCNT est lié à plusieurs facteurs. D'abord, la régression des maladies infectieuses qui affectent de manière disproportionnée les enfants, conduit à l'augmentation de l'espérance de vie chez les adultes et un vieillissement relatif de la population [3]. Ensuite, l’évolution du profil démographique de la population est un facteur important qui influe, dans l'avenir, sur l'incidence des MCNT en Afrique. Enfin, l'urbanisation et le changement des modes de vie liés au développement économique sont étroitement liés à la survenue des MCNT [3, 13, 14]. Ces changements sont relatifs à l'alimentation, l'activité physique, le tabagisme, l'obésité et la consommation d'alcool. Dans les pays développés, environ 90% des nouveaux cas de diabète et 70 à 80% des maladies cardiovasculaires sont imputables au style de vie [3, 15, 16]. Une étude précédente réalisée au Sénégal a montré une fréquence élevée de l'HTA (24,1%), du diabète (9,7%), de l'obésité (16,7%) et de l'insuffisance rénale (22,4%) et la plupart des patients dépistés ignorait leur statut médical [17]. Les MCNT constituent un lourd fardeau économique dans les pays à faible revenus et les ménages pauvres sont les plus affectés [18, 19]. La couverture d'assurance maladie est encore limitée et les frais médicaux sont supportés en grande partie par les familles en général [18, 20, 21]. Ainsi, il apparait urgent de freiner l’évolution croissante des MCNT dans les pays à faible et moyen revenus, et de réduire les charges sociales et économiques qui en découlent [22–24]. Les interventions doivent être accès sur la prise en compte des facteurs de risque tels que l'usage du tabac, la consommation de l'alcool, la sédentarité, et l'alimentation inappropriée [22, 25–27].

L'objectif de l’étude était d’évaluer les facteurs de risque des MCNT afin de d'estimer la prévalence des maladies chroniques non transmissibles et leurs facteurs de risque au niveau des forces armées Sénégalaises.

## Méthodes

Il s'agissait d'une étude transversale descriptive et analytique portant sur les principaux facteurs de risque (comportementaux, physiques et biologiques) des maladies non transmissibles. Les outils des enquêtes STEPS 1, 2 et 3 ont été utilisés. Les militaires sénégalais âgés de 25 à 60 ans, présents au Sénégal au moment de l'enquête, étaient éligibles. Cette population était répartie en quatre (04) strates: 25-34 ans; 35-44 ans; 45-54 ans et 55-60 ans.

L’échantillon a été déterminé à partir d'une proportion initiale de 25% et une précision de 5%. L'effet du plan de sondage a été estimé à 1,5. Ainsi, pour chaque strate un effectif de 432 devrait être enquêté, en dehors de la dernière où la moitié était prévue. La taille de l’échantillon est donc égale à 1513. Une stratification à deux niveaux a été utilisée pour la sélection des unités statistiques.

Le questionnaire STEPS adapté au contexte des Armées a été utilisé [28]. Il présentait une section sur les informations sociodémographiques et comportementaux, une sur les mesures anthropométriques et cardiovasculaires et enfin une sur les paramètres biochimiques. L’étude était autorisée le chef militaire et la participation était libre et volontaire.

Les données ont été saisies avec le logiciel Epi info 6 et analysées à l'aide de R^®^. Cinq facteurs ont permis de déterminer le score de risque cardiovasculaire: fumer quotidiennement de la cigarette, consommer moins de 5 portions de fruits et légumes par jour, avoir moins de trois séances activités physiques intenses ou modérées par semaine, être hypertendu (PAS ≥ 140 mm Hg et/ou PAD ≥ 90 mm Hg ou être sous antihypertenseur) et enfin avoir un IMC ≥ 25 kg/m^2^. Une portion de fruits et légumes correspond à 80 grammes de fruits et légumes. L'estimation était faite par les enquêtés à l'aide de carte. L'activité physique modérée correspond à une séance soutenue pendant 30 minutes et entrainant une augmentation modérée de la fréquence cardiaque. L'activité physique intense est définie par une séance de loisir ou de sport soutenue au moins 30 minutes et entrainant une accélération importante du rythme cardiaque [3, 29].

La combinaison de ces déterminants a permis de déterminer un score en fonction du nombre de facteurs présents. Selon, l'OMS, ceux qui ne présentent aucun facteur de risque sont classés «faible risque cardiovasculaire» et ceux ont au moins trois déterminants, sont classés «risque cardiovasculaire élevé» [1, 3, 29]. Le test de Chi^2^ a été utilisé pour la comparaison des proportions et le seuil de significativité était fixé à 5%.

## Résultats

Ces résultats concernent 1125 militaires enquêtés. L’âge moyen était de 39,7±9,2 ans et la médiane à 40 ans. La plupart des enquêtés étaient des hommes avec un sex-ratio de 28,6. Plus de huit participants sur dix étaient mariés et les célibataires représentaient un peu moins de 2%. Le nombre moyen d'années passées à l’école était de 10,8 ±3,5 ans avec une médiane égale à 11 ans. Un peu plus de la moitié des participants a atteint au moins le niveau secondaire. La consommation quotidienne de cigarette était de 17,3% et ne variait pas significativement entre les catégories d’âges (p = 0,57). L’âge moyen auquel ils ont commencé à fumer est 20,8±4 ans, la médiane était de 20 ans, le minimum de 12 et le maximum 39 ans. Le nombre moyen de cigarettes industrielles consommées quotidiennement était de 9,5±4,7 chez les fumeurs actifs; la médiane était de 10, le minimum de 2 et le maximum de 20 cigarettes (Tableau 1).

**Tableau 1:** Caractéristiques socio-démographiques et cliniques des participants

Variables	Moyenne	Ecart-type
Age (années)	39,7	9,17
Nombre d'années d’étude	10,9	3,46
Age début tabagisme actif (années)	20,8	4,0
Nombre de cigarettes/jour	9,5	4,7
Nombre de portions de fruits et légumes/jour	4,2	11,1
Pression artérielle diastolique (mm Hg)	80,1	10,5
Pression artérielle systolique (mm Hg)	128,9	21,0
Indice de masse corporelle (kg/m^2^)	23,7	3,1

**Figure 1:**
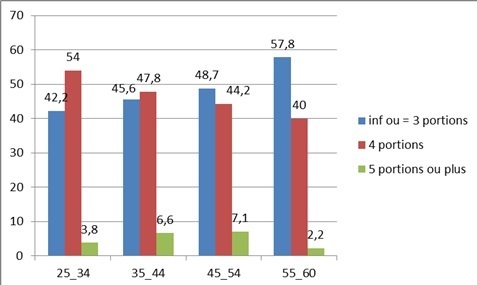
Niveau de consommation de fruits et légumes par catégorie d’âge

La consommation de fruits et légumes, mesurée sur une échelle à trois niveaux, montre que 46% des enquêtés consommaient chaque jour 3 portions ou moins, 48,4% exactement 4 portions et 5,7% cinq portions ou plus. Il n'existe une variation importante entre les différentes catégories d’âge. La Figure 1 illustre le niveau de consommation de fruits et légumes au niveau de chaque strate.

Presque trois enquêtés sur dix avaient une activité physique limitée. Cette proportion était variable selon la catégorie d’âge; élevée chez les 55-60 ans (53,3%) et relativement faible chez les 25-34 ans (17,9%). Le nombre moyen de jours de sport par semaine était de 2,7±1,5 (0-7 jours) (Tableau 2). La proportion d'enquêtés ayant une HTA systolique était de 22,6% (p = 0,002). Elle était moins importante chez les 25-44 ans (19,2%), que chez les 45-60 (29%). L'HTA diastolique suit le même profil avec une fréquence 22% (p = 0,003). Seulement 25 enquêtés étaient sous traitement médical antihypertenseur. La prévalence globale de l'HTA était de 28,4% (p< 0,001). Chez les individus âgés de 25-44ans, un quart était hypertendu contre un tiers chez les plus âgés (Tableau 2).

**Tableau 2:** Répartition des facteurs de risque cardiovasculaire selon la classe d’âge dans la population étudiée (N = 1125)

	Total	25-34 ans	35-44 ans	45-54 ans	55-60 ans	p-value
		n(%)	n(%)	n(%)	n(%)	
Tabagisme actif	194(17,3)	52(15,0)	73(18,5)	62(18,3)	7(15,6)	0,570
**Consommation fruits et légumes**						
≤ 3 portions	517(46,0)	146(42,2)	180(45,6)	165(48,7)	26(57,8)	0,140
4 portions	544(48,4)	187(54,0)	189(47,8)	150(44,2)	18(40,0)	0,044
≥ 5 portions	64(5,7)	13(3,8)	26(6,6)	24(7,1)	1(2,2)	----
**Activité physique**						
***Au travail***						
Limitée	492(43,7)	116(33,5)	153(38,7)	196(57,8)	27(60,0)	< 0,001
Modérée	436(38,8)	141(40,8)	171(43,3)	108(31,9)	16(35,6)	0,012
Intense	267(23,7)	117(33,8)	98(24,8)	48(14,2)	4(8,9)	< 0,001
***Loisir ou Sport***						
Limitée	741(65,9)	203(58,7)	272(68,9)	232(68,4)	34(75,6)	0,006
Modérée	213(18,9)	55(15,9)	72(18,2)	77(22,7)	9(20,0)	0,145
Intense	230(20,4)	107(30,9)	75(19,0)	44(13,0)	4(8,9)	< 0,001
*Activité intense ou modérée*	808(71,8)	284(82,1)	296(74,9)	207(61,1)	21(46,7)	< 0,001
**Hypertension artérielle (HTA)**						
HTA systolique	254(22,6)	63(18,2)	79(20,0)	99(29,2)	13(28,9)	0,002
HTA diastolique	247(22,0)	58(16,8)	82(20,8)	95(28,0)	12(26,7)	0,003
Traitement anti-HTA	25(2,2)	1(0,3)	6(1,5)	13(3,8)	5(11,1)	------
HTA systolo-diastolique	320(28,4)	77(22,3)	105(26,6)	123(36,3)	15(33,3)	<0,001
**Indice de masse corporelle (kg/m** ^**2**^ **)**						
Normal	781(69,4)	311(89,9)	257(65,1)	191(56,3)	22(48,9)	<0,001
Surcharge pondérale	304(27,0)	32(9,2)	120(30,4)	130(38,3)	22(48,9)	<0,001
Obésité	40(3,6)	3(0,9)	18(4,6)	18(5,3)	1(2,2)	-----
Score de risque cardiovasculaire						
0	6(0,5)	1(0,3)	4(1,0)	1 (0,3)	0(0,0)	---
1	129(11,5)	44(12,7)	48(12,2)	35(10,4)	2(4,4)	0,340
2	549(48,8)	191(55,2)	200(50,6)	140(41,4)	18(40)	0,002
3	359(31,9)	100(28,9)	125(31,6)	117(34,6)	17(37,8)	0,360
4	81(7,2)	10(2,9)	18(4,6)	45(13,3)	8(17,8)	----

La surcharge pondérale était fréquente dans l'ensemble (27%) variant considérablement d'une catégorie d’âge à une autre. Elle était retrouvée chez presque la moitié des individus âgés de 55-60 ans, 40% de ceux âgés de 45-54 ans, 30% de ceux âgés de 35-44 ans et moins de 10% des individus âgés de 25-34 ans. La prévalence de l'obésité (IMC ≥ 30kg/m^2^) était faible (3,6%) (Tableau 2). La prévalence du diabète était de 3,0% et seul un patient avait un débit de filtration glomérulaire inférieur à 60 ml/min/1,73m^2^.

**Figure 2:**
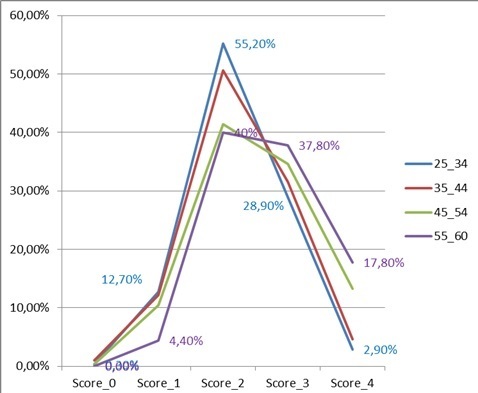
Distribution du score de risque cardiovasculaire en fonction de la catégorie d’âge

La détermination du score de risque a permis de constater que moins de 1% des participants ne présentait aucun facteur de risque, 11,5% avaient seulement un facteur de risque, 49% deux facteurs, 32% trois facteurs et 7% des individus combinaient quatre facteurs de risque. Selon la classification du risque cardiovasculaire de l'OMS, moins de 1% est à risque faible et 39,1% à risque élevé. Tel qu'illustré par le graphique ci-dessous, l'analyse du risque cardiovasculaire montre une variabilité entre les classes d’âge (p < 0,001); la présence d'au moins trois facteurs de risque était de 31,8% et 36,2% respectivement chez les 25-34 ans et les 35-44 ans; de 47,8 et 55,5% chez les 45-54 ans et les 55-60 ans. La Figure 2 illustre le score du risque en fonction de la catégorie d’âge.

## Discussion

Les études épidémiologiques portant sur les facteurs de risque des MCNT sont encore rares en Afrique au Sud du Sahara [3]. Et pour l'essentiel, elles concernent des groupes particuliers. Cette présente étude a été réalisée auprès d'une population sous les drapeaux, âgée entre 25 et 60 ans.

La consommation quotidienne de tabac (17,3%), qui constitue un facteur de risque cardiovasculaire important, était élevée par rapport au seuil de 7% observée chez les travailleurs du secteur privé sénégalais [17]. Cependant, elle est comparable à la proportion observée au Nigéria [30, 31] et inférieure à celle notée en Afrique du Sud [32]. La plus part des participants ont commencé à fumer à l’âge de 20 ans. Probablement le stress professionnel pourrait expliquer l'importance et la constance de cette pratique dans notre étude. L'OMS recommande la consommation journalière de 5 portions ou 400 grammes de fruits et légumes. Dans notre population d’étude, la consommation de fruits et légumes n'est pas adéquate. Cependant, elle pourrait être rapidement améliorée, presque 50% consomment déjà 4 portions par jour.

Comme prévu, le taux de sédentarité chez les militaires est plus faible que chez les travailleurs du secteur privé au Sénégal [17]. Ceci s'explique par le fait dans les armées, le tableau de travail prévoit deux jours de sport par semaine servant parfois à organiser des séances collectifs. En plus, l'activité physique est un critère d’évaluation de la performance dans les armées.

La prévalence de l'HTA observée est à peu près identique à celles trouvée dans d'autres études antérieures [17, 33]. Elle reste préoccupante et en constante augmentation dans les pays développés et surtout ceux en développement [34]. Certaines études montrent des variations importantes ente 6 et 48% selon les pays, et selon le milieu rural ou urbain [3]. Toutefois, certains auteurs prévoient une augmentation de la prévalence de l'HTA au cours des prochaines de 80% et 24% respectivement dans les pays en développement et dans les pays développés [34].

En Afrique sub-Saharienne (ASS), il existe une relation complexe entre l'insuffisance pondérale et le surpoids. Le déficit pondéral est surtout observé chez les enfants et l'excès chez les adultes surtout du genre féminin [3]. Les prévalences de l'obésité et de la surcharge pondérale trouvées dans cette étude sont relativement faibles comparées aux résultats de Seck et al qui rapportaient respectivement 16,7% et 81% chez les travailleurs du secteur privé Sénégalais [17]. Ceci pourrait s'expliquer par un niveau d'activité physique plus élevé dans notre population d’étude composée de militaires.

L'importance du risque cardiovasculaire selon la classification de l'OMS montre la place des MCNT dans notre étude. Les pathologies chroniques cardio-métaboliques et rénales sont en augmentation en ASS du fait d'une fréquence croissante leurs facteurs de risque [3, 13, 35, 36]. Cependant, ces facteurs sont mal quantifiés dans la plus part des pays. En effet, les données sont disparates et pourraient probablement être l'illustration de l’évolution des situations qui se produisent entre les groupes, les régions voire les pays [3]. Les accidents vasculaires cérébraux et les infarctus du myocarde s'observent plus fréquemment chez des sujets jeunes.

La recherche sur les MCNT en ASS a été très négligeable par rapport à celle menée dans les pays développés et la majorité des études publiées ont été réalisées en milieu hospitalier ou clinique. Les études communautaires montrent souvent une forte prévalence des MCNT [3, 37–39]. Il est alors urgent d'agir pour atténuer l’éclosion d'une épidémie de MCNT en Afrique.

Cette étude présente cependant certaines limites liées la population étudiée soit composée d'individus sélectionnés vers l’âge de 20 ans sur la base d'un bon état de santé et bénéficiant d'un suivi médical régulier. Ainsi, elle n'est pas forcément représentative de la population nationale et les résultats ne sont pas directement transposables sur la population générale.

## Conclusion

Cette étude a permis de démontrer l'importance des facteurs de risque des MCNT et surtout de mettre en évidence le risque cardiovasculaire élevé chez les militaires sénégalais, selon la classification de l'OMS. A l'image du VIH/Sida, l’éclosion d'une épidémie de MCNT va constituer également un problème de sécurité et risque d'impacter négativement sur le fonctionnement de l'institution. Certaines politiques en rigueur telle que la prise en charge gratuite des maladies à soins coûteux ne sera plus viable à long terme. Une alternative durable serait la mise en place d'un programme de prévention primaire, accès sur la lutte contre le tabac, la prévention de la surcharge pondérale, et la promotion d'une bonne alimentation. Le service de santé des Armées doit aussi mettre en place un plateau technique pour le dépistage précoce et la prise des urgences cardiovasculaires à tous les niveaux de la chaîne santé.
